# Pilot project aimed at enhancing nursing skills for managing vascular access devices in the oncohematology setting

**DOI:** 10.1177/11297298261424633

**Published:** 2026-03-28

**Authors:** Federico Aula, Beatrice Faccini, Anna Campostano, Francesco Ursino, Damiano Consoli, Filippo Bondielli, Rosina Sapia, Molinari Francesca, Marco Cappellin, Alessandro Fasciolo, Luca Boni, Sergio Bertoglio, Andrea Gambino, Matilde Mannucci

**Affiliations:** 1 Department of Emergency, IRCCS Ospedale Policlinico San Martino, Genova, Italy; 2 Department of Nursing Sciences, IRCCS Ospedale Policlinico San Martino, Genova, Italy; 3 IRCCS Ospedale Policlinico San Martino, Genova, Italy; 4 Stroke Unit, Ospedale Civile Santa Maria degli Angeli, Pordenone, Italy; 5 Unit of Clinical Epidemiology, IRCCS Ospedale Policlinico San Martino, Genova, Italy; 6 Plan 1 Health S.r.l., Amaro, Udine, Italy

**Keywords:** Vascular access device, nursing education, adverse effects, device removal, catheterization, peripheral

## Abstract

**Introduction:**

Venous vascular access devices (VAD) provide a safe route for drug administration but are not free from potential complications and failures. Proper training on their management is essential to reduce these events by ensuring staff adherence to current recommendations and guidelines. Correct management is crucial to ensure safe and effective care. However, the growing use of these devices has been accompanied by an increase in related complications.

**Objectives:**

To evaluate the effectiveness of an online training program (FAD) for nursing staff in reducing VAD failures (PICC, MiniMidline, Midline, PORT, and PICC-port) and improving theoretical knowledge of vascular access management.

**Materials and methods:**

A prospective observational three-phase study was conducted over 14 months (2022–2023) at the Onco-Hematology Department of IRCCS Ospedale Policlinico San Martino, Genoa. A total of 1098 patients undergoing VAD placement were included (532 pre-training and 562 post-training). The primary endpoint was the reduction in device failure, defined as any complication leading to removal. Incidence rates were compared between periods using an adjusted Poisson regression analysis. As a secondary objective, the effect of training on MiniMidline and Midline device performance was evaluated by including the interaction between training and device type in the model. Pre- and post-course test scores were also analyzed.

**Results:**

The overall failure rate did not show a statistically significant reduction (RR 0.87; 95% CI 0.59–1.26; *p* = 0.46). However, a significant reduction in Midline failure risk was observed after training (RR from 5.33 to 2.20; *p* < 0.001). Nurses’ mean test scores significantly improved from 11.00 to 12.14 out of 15 (*p* < 0.001).

**Conclusions:**

The online training program significantly improved nurses’ theoretical knowledge and reduced complications related to Midline devices. However, to achieve a broader impact on VAD-associated complications, structured and continuous training programs are needed.

## Introduction

The use of peripheral and central venous access is a significant aspect in patient care. At the IRCCS San Martino Polyclinic in Genoa, the number of placements of venous access devices (VAD) has been exponentially increasing, mainly for three reasons: (a) a greater tendency among clinicians to use vascular access devices, (b) the simplicity and minimally invasive nature of insertion procedures at the patient’s bedside, (c) the presence of a PICC-Team dedicated to the placement of VADs. However, in parallel with the growing number of insertions, a corresponding increase in complications related to device management has been observed. The most frequent complications are infectious (catheter-related, bloodstream infections, local infections), thrombotic, and mechanical (occlusions, dislocations, and accidental removals).^1^ In many cases, such complications necessitate the removal of the device, an event defined as “device failure.”^2^ At the San Martino Polyclinic, on average, more than three thousand procedures for the implantation of central and peripheral venous devices of short, medium, and long duration are performed annually. Among healthcare workers, there is a widespread perception that in recent years there has been an increase in management complications, particularly infections, a phenomenon that seems to have further intensified during the SARS COV 2 pandemic. The causes of these complications are partly attributed to the shortage of nursing resources, which leads to management difficulties, and partly to the lack of adequate implementation of training and management programs.^3–6^ One of the fundamental pillars to reduce complications related to vascular accesses is “nursing training,” which plays a key role in preparing nurses to align their clinical practice with the most authoritative reference guidelines.^7–10^ One effective strategy to prevent such events is therefore the introduction of continuous training programs on vascular accesses.^11,12^ In light of these considerations, the need to activate a dedicated training pathway for the correct management of vascular accesses was highlighted. The objective of this pathway is to improve management procedures used in clinical practice, with the intent of reducing the rate of complications and failures. This project was initially launched within the onco-hematology unit, a choice motivated by the high number of fragile and/or immunocompromised patients treated in this setting and by the possibility of continuous patient monitoring, since all patients belong to a single unit, thus minimizing data loss.

## Materials and methods

A prospective observational study lasting a total of 14 months was conducted during the period 2022–2023 in the Onco-hematology department of the IRCCS San Martino Polyclinic Hospital in Genoa.

The study was divided into three phases:

Phase I: a 6-month observational period,
Phase II: a 2-month educational intervention, consisting of a distance learning course (FAD) for all nurses belonging to the area under examination regarding the correct management of vascular access devices,
Phase III: a second 6-month observational phase, conducted using the same methods as Phase I, to evaluate the effectiveness of the FAD course.

This study was reported in accordance with the STROBE (Strengthening the Reporting of Observational Studies in Epidemiology) guidelines, and the corresponding checklist is provided as Supplemental Material.

Patients included in Phases I and III were identified among all subjects undergoing placement of a VAD (PICC, MiniMidline and Midline, PORT, and PICC-port) in two different time periods, before and after the training intervention. For each patient, only the first failure was recorded, that is, a complication leading to device removal. During the follow-up periods, data were collected on all central venous access devices (PICC, PORT, and PICC-Port) and peripheral devices (Midline and Mini-Midline).

In Phase II, all healthcare professionals in the Onco-Hematology Department involved in patient management during Phases I and III participated in the mandatory training course. The course, organized by nurses specialized in vascular access, was held in FAD mode (Corporate Distance Training) and was divided into six modules:

Module I: Devices present in the Company: indications for use
Module II: Request for company application, assessment scales of venous assets
Module III: Best practice: guidelines and correct execution of blood culture
Module IV: Infectious complications
Module V: Mechanical complications
Module VI: Dressings and fixation systems present in the company

The course was made available within 1 month after the conclusion of Phase I and remained accessible to staff for 2 months. To evaluate the knowledge level of nursing staff, the course was accompanied by a pre-test and post-test, specifically designed for this study and consisting of 15 multiple-choice questions (Supplemental Material).

All collected data were stored in a database and processed anonymously, in compliance with current Italian regulations and the ethical principles outlined in the Declaration of Helsinki. The study protocol was approved by the Institutional Ethics Committee of the IRCCS Policlinico San Martino (n.126/2022-DBid12211) and written consent was obtained from all participants.

### Study objectives

The primary objective was to evaluate the effectiveness of the training intervention by analyzing the possible reduction in the device failure rate. The secondary objective was to assess the impact of the training intervention on nurses’ knowledge of the topics addressed during the course, as measured by the ad hoc pre and post training tests.

### Statistical analysis

Patient data collected at baseline and considered in the analysis included, in addition to the type of VAD namely PICC, Midline (a category including MiniMidline and Midline devices), PORT and PICC-port, sex, age at the time of VAD insertion (classified into four categories: 20–57, 58–65, 66–73, 74–83 years), care setting (ordinary hospitalization or day hospital), and type of pathology (oncological and non-oncological). Continuous variables were summarized using medians values and interquartile ranges (IQR), while categorical variables were described through absolute and relative frequencies (percentages). For each category of the analyzed factors, a catheter failure rate (CFR) was calculated as the ratio between the number of removals and the observation time, expressed in person-days accumulated during the follow-up period. A corrected Poisson regression analysis^13^ was also applied to evaluate the effect of training on the frequency of removals; results were reported as removal rate ratios (RR) and their 95% confidence intervals (95% CI). Furthermore, a Poisson model including an interaction term between device type and participation in the training course was used to estimate the effect of the intervention on Midline devices compared to other VADs. Data collected from healthcare workers concerned only responses to test questions; no sociodemographic or work-related variables were included in the questionnaires. Final test scores obtained before and after the training event were described using mean and standard deviation and then compared using a paired-samples *t*-test. A *p*-value ⩽0.05 was considered statistically significant. All statistical analyses were performed using Stata software (Stata Corp. Stata: Release 17. Statistical Software. College Station, TX. 2021).

## Results

**Figure 1.**
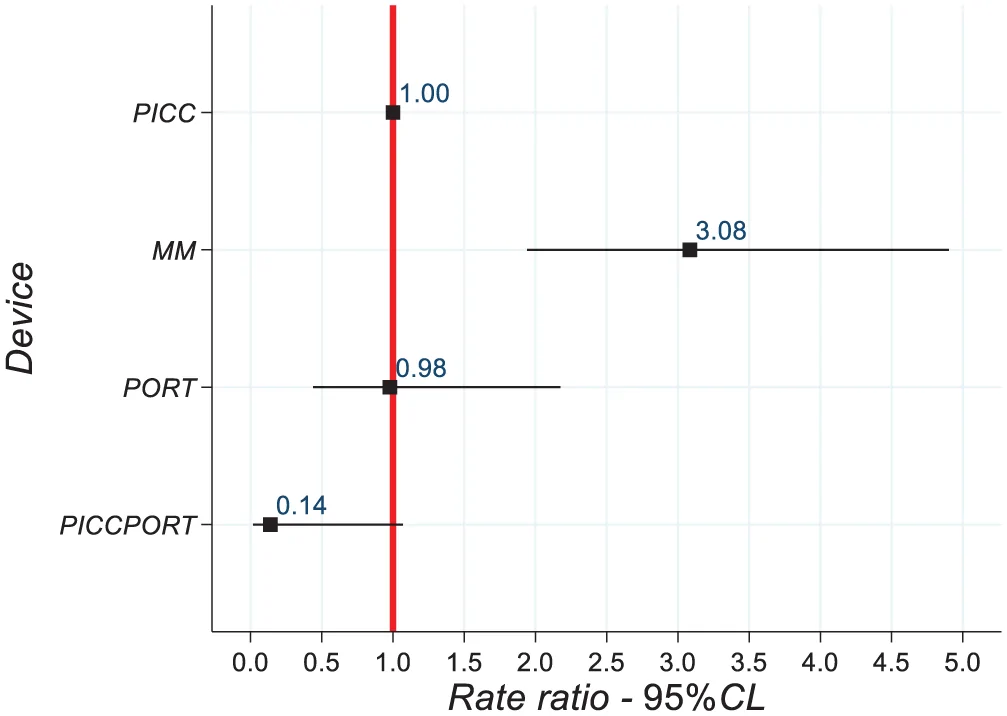
Association between device type and risk of failure: RR and 95% CL, obtained through multivariable Poisson regression with robust standard errors. MM: Midline/MiniMidline; PICC: peripherally inserted central catheter; Others: PICC, PORT, PICCPORT; RR: rate ratio; 95% CL: 95% confidence limits for RR.

A total of 1098 patients undergoing VAD placement were included in the study: 532 in the pre-training intervention period and 562 in the post-training intervention period. Table 1 presents the characteristics of the total patients and those divided into the two enrollment periods. In particular, the overall median age was 64 years (IQR 54–73), showing a prevalence of elderly patients (69% in the 58–83 age range), with very similar values in the two subgroups. Women were generally more frequent than men (58.7% vs 41.3%), as well as in the pre- (62.6% vs 37.4%) and post-training periods (55.1% vs 44.9%). Nearly all patients (99.4%) had oncological pathology. The most frequently inserted devices were PICCs (49.3%), followed by PORTs (20.6%), Midlines (18.1%), and PICC-ports (12.0%). When comparing the two study periods, there was an increase in the use of PICC and Midline devices, which rose respectively from 45.7% to 52.7% and from 16.0% to 20.1%, and a decrease in PORT and PICC-port devices, which dropped respectively from 23.7% to 17.7% and from 14.7% to 9.5%. Median follow-up times were 1.8 months in the first study period (IQR 0.6–3.7; min 0.03; max 6.1) and 2.0 months in the second (IQR 0.7–3.6; min 0.03; max 6.0). A total of 123 complications were recorded, of which 109 were failures: 49 in the pre-training period and 60 in the post-training period. After adjustment for the main confounding factors, a slight reduction in failure risk of about 13% was observed (RR 0.87, 95% CI 0.59–1.26), which was not statistically significant (*p* = 0.46) between patient groups before and after training for all devices (Table 2). Analysis of the entire sample revealed a higher failure rate in the case of Midline device use (CFR 3.86, 95% CI 2.29–6.51), with a higher risk of failure compared to all other devices even after adjustment for major confounders (RR 3.08, 95% CI 1.94–4.90; Table 2 and Figure 1).

**Table 1.** Patients’ characteristics in pre and post nursing training groups.

Characteristics	Pre		Post		Total	
	*N*	%	*N*	%	*N*	%
Age (median, IQR)	64 (54–72)		64 (55–73)		64 (54–73)	
20–57	169	31.8	172	30.4	341	31.1
58–65	125	23.5	122	21.6	247	22.5
66–73	129	24.2	133	23.5	262	23.9
74–83	109	20.5	139	24.6	248	22.6
Gender						
M	199	37.4	254	44.9	453	41.3
F	333	62.6	312	55.1	645	58.7
Care setting						
Hospitalization	202	38.0	266	47.0	468	42.6
Day hospital	330	62.0	300	53.0	630	57.4
Pathology						
Oncological	525	98.7	566	100	1091	99.4
Non-oncological	7	1.3	0	0	7	0.6
Device						
PICC	243	45.7	298	52.7	541	49.3
MM	85	16.0	114	20.1	199	18.1
PORT	126	23.7	100	17.7	226	20.6
PICCPORT	78	14.7	54	9.5	132	12.0
Total	532	100	562	100	1098	100

*N* %: absolute/relative frequency; IQR: inter-quartile range; MM: Midline/MiniMidline; PICC: peripherally inserted central catheter.

This table provides a descriptive summary of the study population; no hypothesis testing was conducted.

**Table 2.** Multivariable Poisson regression results.

Variable	Multivariate model*				
	CFR	95% CL	RR	95% CL	*p*
Training					
Before	1.30	0.90–1.88	1.00	(Rif.)	0.46
After	1.12	0.78–1.62	0.87	0.59–1.26	
Device					
PICC	1.25	0.89–1.76	1.00	(Rif.)	<0.001
MM	3.86	2.29–6.51	3.08	1.94–4.90	
PORT	1.22	0.64–2.33	0.98	0.44–2.17	
PICCPORT	0.17	0.02–1.26	0.14	0.02–1.07	
Total	1.47	1.22–1.77			

MM: Midline/MiniMidline; PICC: peripherally inserted central catheter; CFR: catheter failure rate per year per 1000 patients; RR: rate ratio; 95% CL: 95% confidence limits for CFR/RR; Rif.: reference category; *p*: probability level associated with the overall likelihood-ratio test for each variable.

* Adjusted for care setting, gender, age.

**Figure 2.**
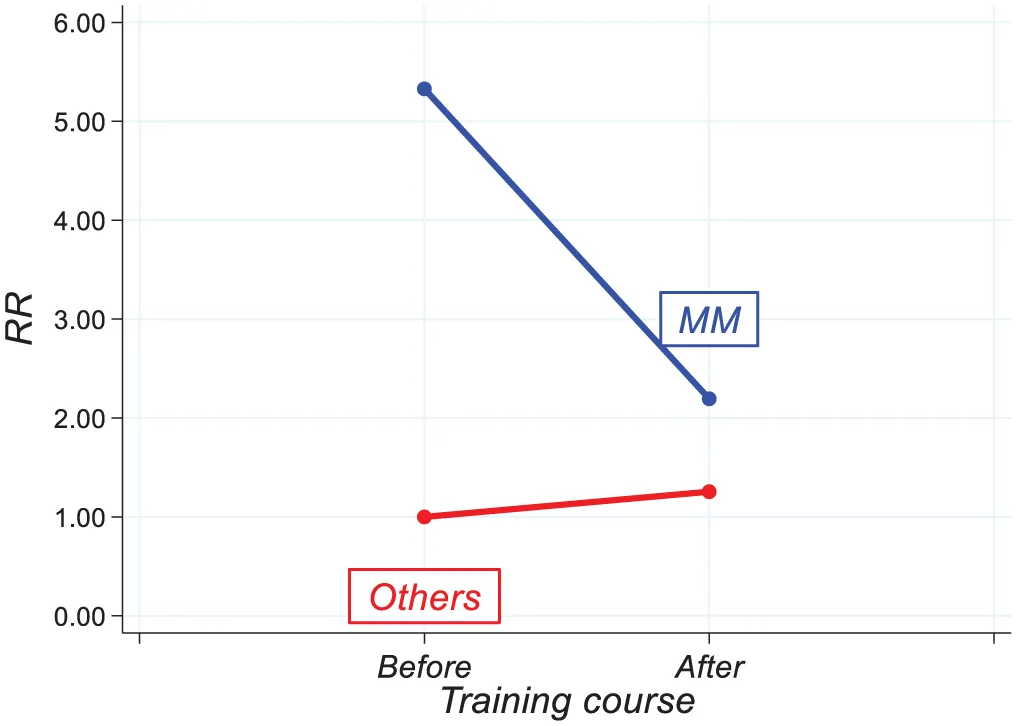
Interaction between training and device type in the risk of failure: variation in risk (RR) before and after training course. MM: Midline/MiniMidline; Others: PICC, PORT, PICCPORT; RR: rate ratio.

The interaction between training and device type (Table 3, Figure 2) showed a significant reduction in the failure risk associated with Midline devices after the training course. Before training, the RR for subjects with Midline compared to those with other devices was 5.33 (95% CI 2.92–9.72), which decreased to 2.20 (95% CI 1.11–4.36) after training, with *p* < 0.001. This suggests an effect of training in reducing the failure risk associated with these devices. Regarding the knowledge assessment, a total of 99 healthcare professionals, all those managing VADs in Phases I and III, completed the questionnaires. The analysis showed a significant increase in mean scores after the training intervention: from 11.00 pre-training to 12.14 post-training. The mean difference (Md 1.14, 95% CI 0.65–1.63) was statistically significant (*p* < 0.001), indicating improved knowledge following the training intervention (Table 4).

**Table 3.** Interaction between device type and training course^*^.

Training	Device	RR	95% CL	*p*
Before	Others	1.00	(Rif.)	0.001
	MM	5.33	2.92–9.72	
After	Others	1.26	0.79–2.00	
	MM	2.20	1.11–4.36	

MM: Midline/MiniMidline; Others: PICC, PORT, PICCPORT; RR: rate ratio; 95% CL: 95% confidence limits for RR; Rif.: reference category; *p*: probability level associated with the likelihood-ratio test, interaction term between training and device type.

* Adjusted for care setting, gender, age.

**Table 4.** Variation in mean score obtained before and after training.

*N*	Mean		Md	95% CL	*p*
	Before	After			
99	11.00	12.14	1.14	0.65–1.63	0.001

Md: mean difference; *p*: probability level associated with the paired samples *t*-test.

## Discussion

Medicine is a constantly evolving science that requires all healthcare professionals to engage in continuous education in order to keep pace with new knowledge and techniques. Training initiatives for healthcare workers in these contexts plays a fundamental role, as it prepares nurses and physicians to meet standards and guidelines based on the best available evidence, thereby ensuring safe and effective practice.^14^ In the clinical context, nurses’ education and knowledge regarding the management and complications of vascular access devices must be considered a key factor. Inadequate training can partially contribute to non-standardized practices, potentially resulting in a higher incidence of mechanical and infectious complications and a consequent reduction in the effectiveness and safety of vascular access devices. Literature has reported that oncology nurses may sometimes exhibit an unsatisfactory level of knowledge in the field of vascular access.^14^ The present study aimed to optimize the clinical management of these devices through a distance learning course (FAD) designed to enhance both their efficacy and safety in the onco-hematology setting. In our experience, the single training event had a positive impact on the theoretical knowledge level of nurses. However, although a reduction in the overall rate of vascular access complications was observed, this difference did not reach statistical significance. The most significant effect was observed only in the subgroup of Midline devices. This result suggests that specific training for nurses had a relevant impact on the management of more complex devices such as Midlines, which, both in our study and in the literature,^2^ appear in oncological settings to be more frequently associated with complications compared to PICCs. The apparent discrepancy between improved knowledge and the limited reduction in complications could be attributed to the initially low baseline level of awareness and expertise regarding vascular access nursing principles. For this reason, a single FAD course may be insufficient to translate knowledge into sustained clinical improvement. Moreover, although practical skills are considerable importance, their assessment was not included among the objectives of this study, which primarily aimed to evaluate the impact of distance learning on clinical outcomes. It should also be noted that the absence of in-person training would, in any case, have made a direct and reliable evaluation of practical competencies methodologically challenging.

### Limitations

This study has several limitations that should be considered when interpreting the results. First, the educational intervention consisted of a single online training course, and we did not assess the long-term retention of knowledge or actual behavioral changes in clinical practice, which may limit the sustainability of the observed improvements in device management. Second, the study did not include an objective evaluation of practical skills acquisition, focusing instead on theoretical test scores, which may not fully reflect nurses’ competence in hands-on catheter care or adherence to procedural standards. Third, this study was conducted at a single-center in a specific onco-hematology unit, which may limit generalizability of the findings to other clinical settings. Finally, as is common with many observational designs, there may have been unmeasured confounding factors that could have influenced catheter failure rates independently of the training intervention. Acknowledging these limitations provides appropriate context for interpreting the current findings and suggests directions for future research. Future studies could benefit from a multicenter design, the incorporation of multimodal educational approaches (such as online training combined with hands-on exercises), and longer follow-up periods.

## Conclusions

Training programs focused on vascular access management are essential to ensure proper handling and maintenance of these devices. However, isolated training events are unlikely to produce lasting improvements in clinical outcomes. Structured, continuous, and long-term educational programs are therefore required to ensure the best clinical outcomes. The results of this study indicate that targeted online training can significantly reduce complication rates for specific types of devices, particularly peripheral MiniMidline and Midline catheters, which have been more recently introduced into clinical practice and are characterized by more complex management. These findings suggest that device-specific training may be particularly effective in settings where baseline experience and procedural standardization are still limited. Future educational strategies should integrate online theoretical modules with hands-on activities and periodic competency assessments in order to improve and standardize nursing practices across different vascular access devices.

## Supplemental Material

sj-docx-1-jva-10.1177_11297298261424633 – Supplemental material for Pilot project aimed at enhancing nursing skills for managing vascular access devices in the oncohematology setting Supplemental material, sj-docx-1-jva-10.1177_11297298261424633 for Pilot project aimed at enhancing nursing skills for managing vascular access devices in the oncohematology setting by Federico Aula, Beatrice Faccini, Anna Campostano, Francesco Ursino, Damiano Consoli, Filippo Bondielli, Rosina Sapia, Molinari Francesca, Marco Cappellin, Alessandro Fasciolo, Luca Boni, Sergio Bertoglio, Andrea Gambino and Matilde Mannucci in The Journal of Vascular Access
