# Supplementary material for: Pilot project aimed at enhancing nursing skills for managing vascular access devices in the oncohematology setting
Source: J Vasc Access. 2026 Mar 28;27(5):2079–84. doi: 10.1177/11297298261424633 (PMC13547684; doi:10.1177/11297298261424633)
Supplement: Supplementary file 1 [file sj-docx-1-jva-10.1177_11297298261424633.docx]

**Pre/Post Test – Vascular Access Devices (VAD) –** Original version

1. **Che cosa è un MIDLINE?**
   a) Un dispositivo venoso periferico la cui lunghezza è compresa fra 15-25 cm^§^
   b) Un dispositivo venoso periferico la cui lunghezza è compresa fra 6-15 cm
   c) Un dispositivo venoso centrale
2. **Che cosa è una cannula lunga?**
   a) Un dispositivo venoso periferico la cui lunghezza è compresa fra 6-15 cm
   b) Un dispositivo venoso periferico a breve termine
   c) Entrambe le precedenti^§^
3. **Cosa è un catetere a breve termine?**
   a) Dispositivo vascolare la cui durata deve essere compresa in massimo 15 giorni
   b) Dispositivo vascolare la cui durata deve essere compresa in massimo 29 giorni^§^
   c) Dispositivo vascolare la cui durata deve essere compresa in massimo 45 giorni
4. **Quando è possibile dimettere un paziente con un VAD a breve termine?**
   a) Mai^§^
   b) Tutte le volte in cui serve a domicilio
   c) Nessuna delle precedenti
5. **Quale disinfettante per antisepsi cutanea è efficace in minor tempo?**
   a) Iodopovidone su base acquosa
   b) Clorexidina 2% in IPA al 70%^§^
   c) Ipoclorito di sodio
6. **Cosa si intende per CRBSI?**
   a) Infezione del sangue
   b) Infezione del catetere
   c) Infezione correlata alla presenza di catetere vascolare^§^
7. **Cosa si intende per complicanza precoce?**
   a) Complicanze intraprocedurali o entro le 24 ore^§^
   b) Complicanze pre-impianto
   c) Complicanze legate alla patologia del paziente candidato al posizionamento
8. **Prima di collegare la linea infusionale all’hub del catetere cosa è raccomandato fare?**
   a) Accertarsi che il deflussore sia idoneo
   b) Disinfettare la porta di accesso al dispositivo^§^
   c) Eliminare NFC e connettersi direttamente all’hub
9. **Qual è la pratica raccomandata per il lavaggio dei dispositivi vascolari?**
   a) Start and Stop^§^
   b) Flusso laminare
   c) Nessuna pratica
10. **La medicazione con garza post-impianto dopo quanto deve essere sostituita?**
    a) 24 ore post-impianto^§^
    b) 12 ore post-impianto
    c) 72 ore post-impianto
11. **Perché è raccomandato utilizzare pellicole trasparenti in poliuretano per la medicazione dell’Exit-Site?**
    a) Per visualizzare l’Exit-Site
    b) Sono altamente traspirabili
    c) Entrambe le risposte^§^
12. **Quando si esegue un’emocoltura?**
    a) Prima della somministrazione dell’antibioticoterapia^§^
    b) Dopo la somministrazione dell’antibioticoterapia
    c) Indifferentemente o prima o dopo l’antibioticoterapia
13. **Quanti cc di sangue, per il paziente adulto, sono consigliati per avere un riscontro significativo dell’emocoltura?**
    a) Tra 2 cc e 5 cc
    b) Tra 3 cc e 6 cc
    c) Tra 8 cc e 10 cc^§^
14. **Quali segni possono segnalare una trombosi dell’arto ospite di dispositivo vascolare?**
    a) La presenza di circoli collaterali sia sull’arto che su collo e torace
    b) La presenza di edema dell’arto
    c) Sono entrambi segni di complicanza^§^
15. **Per il lavaggio del PICC quale soluzione è raccomandato utilizzare?**
    a) Urokinasi
    b) Citrato
    c) NaCl (cloruro di sodio) ^§^

^§^ Correct answer

**Pre/Post Test – Vascular Access Devices (VAD) –** English version

1. **What is a MIDLINE?**
   a) A peripheral venous device with a length between 15-25 cm^§^
   b) A peripheral venous device with a length between 6-15 cm
   c) A central venous device
2. **What is a long cannula?**
   a) A peripheral venous device with a length between 6-15 cm
   b) A short-term peripheral venous device
   c) Both of the above^§^
3. **What is a short-term catheter?**
   a) A vascular device intended to be used for a maximum of 15 days
   b) A vascular device intended to be used for a maximum of 29 days^§^
   c) A vascular device intended to be used for a maximum of 45 days
4. **When is it possible to discharge a patient with a short-term VAD?**
   a) Never^§^
   b) Whenever it is needed at home
   c) None of the above
5. **Which disinfectant is effective in the shortest time for skin antisepsis?**
   a) Povidone-iodine in aqueous solution
   b) 2% Chlorhexidine in 70% isopropyl alcohol^§^
   c) Sodium hypochlorite
6. **What does CRBSI mean?**
   a) Blood infection
   b) Catheter infection
   c) Catheter-Related Bloodstream Infection^§^
7. **What is meant by an early complication?**
   a) Intra-procedural or within 24 hours complications^§^
   b) Pre-implantation complications
   c) Complications related to the underlying condition of the patient
8. **Before connecting the infusion line to the catheter hub, what is recommended?**
   a) Ensure the infusion set is suitable
   b) Disinfect the access port of the device^§^
   c) Remove the NFC and connect directly to the hub
9. **What is the recommended practice for flushing vascular devices?**
   a) Start and Stop technique^§^
   b) Laminar flow
   c) No specific practice
10. **When should the post-implant gauze dressing be changed?**
    a) 24 hours after implantation^§^
    b) 12 hours after implantation
    c) 72 hours after implantation
11. **Why is it recommended to use transparent polyurethane dressings for the Exit-Site?**
    a) To visualize the Exit-Site
    b) They are highly breathable
    c) Both answers^§^
12. **When should blood cultures be performed?**
    a) Before administering antibiotic therapy^§^
    b) After administering antibiotic therapy
    c) Either before or after antibiotic therapy
13. **How many cc of blood are recommended for an adult to ensure meaningful blood culture results?**
    a) Between 2 cc and 5 cc
    b) Between 3 cc and 6 cc
    c) Between 8 cc and 10 cc^§^
14. **What signs may indicate thrombosis in the limb with a vascular device?**
    a) Presence of collateral circulation in the limb, neck, or chest
    b) Swelling (edema) of the limb
    c) Both are signs of complication^§^
15. **Which solution is recommended for flushing a PICC line?**
    a) Urokinase
    b) Citrate
    c) Sodium chloride (NaCl) ^§^

^§^ Correct answer

**STROBE Checklist**

| **Section** | **STROBE Item** | **Study Response** |
| --- | --- | --- |
| **Title and Abstract** | **1a. Study design in title/abstract** | Study design (prospective observational study) is clearly stated in the title and abstract. |
|  | **1b. Abstract summary** | Abstract summarizes background, objectives, methods, results, and conclusions, including device-specific outcomes and P-values. |
| **Introduction** | **2. Background/rationale** | Background provided on VAD complications and importance of nursing training. Rationale for online FAD course described. |
|  | **3. Objectives** | Primary objective: evaluate effectiveness of training on VAD failures; Secondary objective: evaluate impact on nurses’ theoretical knowledge. |
| **Methods** | **4. Study design** | Prospective observational three-phase study design (Phases I, II, III) clearly described. |
|  | **5. Setting** | Conducted in the Onco-Hematology Department of IRCCS Ospedale Policlinico San Martino, Genoa, Italy. |
|  | **6. Participants** | 1,098 patients undergoing VAD placement included (532 pre-training, 562 post-training). Inclusion/exclusion criteria described. |
|  | **7. Variables** | Primary outcome: device failure (any complication leading to removal). Secondary outcome: nurses’ knowledge via pre/post-test. Patient demographics and device type collected. |
|  | **8. Data sources/measurement** | Hospital records for VAD outcomes; 15-item pre/post-test for nursing knowledge. |
|  | **9. Bias** | Potential confounders discussed. Single-center design and observational nature acknowledged. |
|  | **10. Study size** | All eligible patients during study periods included. No formal sample size calculation (pilot study). |
|  | **11. Quantitative variables** | Device failure rates (CFR), removal rate ratios (RR), test scores. Medians, interquartile ranges, percentages used. |
|  | **12. Statistical methods** | Poisson regression for device failures, including interaction between training and device type; paired t-test for test scores; P-values reported as per journal guidelines. |
| **Results** | **13. Participants** | Flow of patients detailed; Table 1 shows demographics and device distribution. |
|  | **14. Descriptive data** | Patient age, sex, pathology type, care setting, and VAD type reported. Median follow-up times provided. |
|  | **15. Outcome data** | 123 complications, 109 failures; overall CFR, RR with 95% CI and P-values provided. |
|  | **16. Main results** | Significant reduction in Midline failure post-training (RR 5.33 → 2.20, P<.001). Overall VAD failures not significant (RR 0.87, P=.46). Test scores improved from 11.00 → 12.14 (P<.001). |
|  | **17. Other analyses** | Interaction analyses between device type and training conducted for MiniMidline and Midline. |
| **Discussion** | **18. Key results** | Improved theoretical knowledge and reduced complications for Midline devices; single training may be insufficient for broad impact. |
|  | **19. Limitations** | Single-center, one-time online course, no long-term follow-up, no practical skills assessment, potential unmeasured confounders. Discussed in Limitations section. |
|  | **20. Interpretation** | Findings interpreted in context of literature; clinical implications and device-specific training effectiveness highlighted. |
|  | **21. Generalizability** | Limited due to single-center design; recommendations for multicenter studies and multimodal educational programs provided. |
| **Other Information** | **22. Funding** | "The authors received no financial support for the research, authorship, and/or publication of this article." |
|  | **23. Conflict of interest** | "The authors declared the following potential conflicts of interest with respect to the research, authorship, and/or publication of this article: Sergio Bertoglio is Chief Medical Officer of Plan1 Health s.r.l., Amaro (UD), Italy." |
